# Are Phe-Free Protein Substitutes Available in Italy for Infants with PKU All the Same?

**DOI:** 10.3390/nu16010030

**Published:** 2023-12-21

**Authors:** Elvira Verduci, Martina Tosi, Chiara Montanari, Mirko Gambino, Francesca Eletti, Alessandra Bosetti, Margherita Di Costanzo, Maria Teresa Carbone, Giacomo Biasucci, Laura Fiori, Gianvincenzo Zuccotti

**Affiliations:** 1 Metabolic Diseases Unit, Department of Pediatrics, Vittore Buzzi Children’s Hospital, University of Milan, 20154 Milan, Italy; 2 Department of Health Sciences, University of Milan, 20146 Milan, Italy; martina.tosi@unimi.it; 3 Department of Pediatrics, Vittore Buzzi Children’s Hospital, University of Milan, 20154 Milan, Italy; chiara.montanari@unimi.it (C.M.); mirko.gambino@unimi.it (M.G.); francesca.eletti@unimi.it (F.E.); alessandra.bosetti@asst-fbf-sacco.it (A.B.); laura.fiori@asst-fbf-sacco.it (L.F.); gianvincenzo.zuccotti@unimi.it (G.Z.); 4 Department of Biomedical and Clinical Science, University of Milan, 20157 Milan, Italy; 5 U.O.C. Pediatrics and Neonatology, Guglielmo da Saliceto Hospital, 29121 Piacenza, Italy; m.dicostanzo@ausl.pc.it (M.D.C.); g.biasucci@ausl.pc.it (G.B.); 6 Department of Medicine and Surgery, University of Parma, 43126 Parma, Italy; 7 UOSD Metabolic Diseases, AORN Santobono-Pausilipon, 80122 Naples, Italy; mt.carbone@santobonopausilipon.it

**Keywords:** phenylketonuria, infant nutrition, Phe-free protein substitutes, docosahexaenoic acid, arachidonic acid, biotics, non-communicable diseases prevention

## Abstract

Breastfeeding or standard infant formulas, alongside phenylalanine (Phe)-free protein substitutes, constitute the dietary management for infants with PKU to guarantee protein requirements are met in compliance with metabolic tolerance. This work aims to analyse the nutritional composition of Phe-free infant protein substitutes, in terms of macronutrients, micronutrients and functional components, available for PKU dietary management in Italy. A total of seven infant Phe-free protein substitutes were included in this review, six powder and one liquid. A second analysis was conducted to compare them to the composition of formulas intended for healthy infants, taking into consideration the Commission Delegated Regulation (EU) 2016/127 and Commission Delegated Regulation (EU) 2016/128 for micronutrients. The analysis revealed heterogeneity among protein substitutes suitable for infants with PKU. The energy and protein equivalents (P.Eq.) content are different; all of the substitutes contain docosahexaenoic acid (DHA) and arachidonic acid (ARA), while eicosapentaenoic acid (EPA), fructo-oligosaccharides (FOS), galacto-oligosaccharides (GOS), human milk oligosaccharides (HMOs) and nucleotides are not present in all the substitutes. More attention should be paid to these infant products to ensure metabolic control of PKU, and also promote proper growth, cognitive neurodevelopment, favourable gut microbiota composition, and immune system health, while reducing the risk for non-communicable diseases (NCDs).

## 1. Introduction

Phenylketonuria (PKU), OMIM: 261600, is an autosomal recessive disorder of phenylalanine metabolism characterized by high phenylalanine (Phe) blood concentrations that cause brain dysfunction and other clinical manifestations [1]. This condition represents the most widespread congenital metabolic disorder of protein metabolism. The prevalence of PKU is currently around 1:10,000 in Europe, with prevalence peaks in some countries, such as Italy, where the incidence is around 1:3300. If not identified via newborn screening and if not treated in the preclinical phase, PKU can lead to various clinical manifestations, such as psychomotor retardation, alterations in cognitive functions, behavioural abnormalities, seizures, pyramidal and extrapyramidal signs, dysmorphic notes, white matter lesions identified with brain magnetic resonance (MRI), abnormal electroencephalogram (EEG) tracing and eczema [2]. Currently, the mainstay of PKU therapy is represented by dietary treatment, with a low and controlled intake of Phe. Dietary management is based on three principles: natural protein restriction, integration with Phe-free protein substitutes (PS) and consumption of low-protein food products. The extent of protein restriction depends on the required amount of Phe for protein synthesis (e.g., age-dependent growth and balance between anabolism and catabolism in periods of illness) and on Phe tolerance, which depends on the severity of the phenylalanine hydroxylase (PAH, 612349) deficiency [3,4]. The remaining amount of the protein requirement must be replaced by Phe-free amino acid substitutes. A strict low-Phe and low-natural protein diet is low in alpha-linolenic acid (ALA), arachidonic acid (ARA), eicosapentaenoic acid (EPA) and docosahexaenoic acid (DHA), as well as in vitamins and minerals such as calcium, iron, vitamin D and B vitamins. Therefore, the supplementation of these compounds is extremely important in cases where they are not already added in the amino acid substitutes [3]. Over the years, more and more attention has been paid to the quality of protein substitutes, leading to the production of better products in terms of nutritional and functional composition, absorption kinetics and palatability, playing a fundamental role in the nutrition of PKU patients since they account for about 50–80% of the total protein intake [3].

### 1.1. Infant Nutrition in PKU

The period of infancy, from birth to 1 year of age, is characterised by special nutritional needs. Meeting the requirements for energy and nutrient amounts per kilogram of body mass and for age leads to adequate growth rate, appropriate synthesis and deposition of body tissues, and also supports optimal immune and central nervous system (CNS) development, also through the gut microbiota influence, with a long-term impact on the health of the subject [5], as highlighted by the Developmental Origins of Health and Disease (DOHaD) Hypothesis, concerning how the perinatal environment, together with early-life nutrition, can impact foetal and adult life [6]. The composition of breast milk, which represents the first infant nutritional source, can satisfy the nutritional needs of infants to support growth and body functions [7]. Compared with formula-fed infants, breast-fed infants present better neurological development, reduced risk for gastrointestinal and respiratory infections, as well as of developing atopy and diabetes in future life [8,9]. When breastfeeding is not possible through the first year of life, infant formulas are needed, aimed at satisfying infants’ nutritional needs until the introduction of adequate complementary nutrition [10]. The main European guidelines on PKU [3] recommend mother’s milk, containing only 46 mg/100 mL of Phe, instead of standard infant formula use, due to DHA and long-chain polyunsaturated fatty acids (LC-PUFAs) content, together with other non-nutritional bioactive and functional compounds. In the management of PKU infants, it is necessary to integrate mother’s milk or standard infant formulas with Phe-free protein substitutes to guarantee that protein requirements are met in compliance with the metabolic tolerance of the infant [3,11,12].

### 1.2. European Regulation for Phe-Free Protein Substitutes for Infants with PKU

The nutritional composition of infant and follow-on formulas used in the European Union (EU) must satisfy the nutritional requirements of infants reported in the Commission Delegated Regulation (EU) 2016/127 of 25 September 2015 supplementing Regulation (EU) No 609/2013 of the European Parliament and of the Council [13]. It defines the specific requirements about composition and provision of information for infant and follow-on formula, an addition to Regulation (EU) No. 609/2013 of the European Parliament and of the Council of 12 June 2013 [10]. Moreover, the products shall comply with the provisions of Regulation (EU) No. 1169/2011 of the European Parliament and of the Council of Council of 25 October 2011 [14] establishing rules on the provision of food information. In the EU, protein substitutes for infants with PKU are considered Foods for Special Medical Purposes (FMSPs) and, according to the Italian Guidelines on Foods for Special Medical Purposes (FMSPs), they are classified as nutritionally incomplete because of the absence of Phe. Infant Phe-free protein substitutes must satisfy the nutritional requirements reported in the Commission Delegated Regulation (EU) 2016/128 of 25 September 2015 supplementing Regulation (EU) No 609/2013 of the European Parliament and of the Council [15] regarding the specific composition and information requirements for FMSPs. Moreover, alike infant formulas, protein substitutes for infants with PKU shall comply with Regulation (EU) No 1169/2011 of the European Parliament and of the Council and Regulation (EU). It is important to highlight that in the Commission Delegated Regulation (EU) 2016/128 it is stated that these FMSPs, developed to satisfy the nutritional requirements of infants with PKU, shall comply with Commission Delegated Regulation (EU) 2016/127 apart from vitamins and minerals substances, whose contents are specifically laid down by Commission Delegated Regulation (EU) 2016/128. Infant Phe-free protein substitutes must comply with the nutritional requirements reported in Commission Delegated Regulation (EU) 2016/127 for infant and follow-on formulas apart from vitamins and minerals that are regulated by Commission Delegated Regulation (EU) 2016/128. The specific compositional requirements for Phe-free protein substitutes for infants with PKU are reported in Appendix A.

## 2. Aims and Methods

This work aims to analyse the differences in terms of macronutrients, micronutrients and other functional components among the infant protein substitutes, liquid, or powder, currently available for PKU dietary management in Italy; secondarily, to compare the nutritional composition of these products to those intended for healthy infants. In this review, semi-solid protein substitutes specifically designed for complementary feeding were excluded. From April to October 2023, researchers have requested the companies producing infant Phe-free PS for PKU under 1 year of age available in Italy to send the updated and detailed nutritional composition data of these products. A total of seven infant Phe-free protein substitutes were included in the review, six powder and only one liquid. When expressed per 100 g, the nutritional composition of each protein substitute has been transformed into 100 mL of liquid, according to the dilution recommended by companies (Table 1). A second analysis allowed us to compare the nutritional values expressed per 100 kcal of product with those reported in the Commission Delegated Regulation (EU) 2016/127 for healthy infant formulas. For vitamin and mineral substances solely, the values considered for the comparison were those reported in the Commission Delegated Regulation (EU) 2016/128 concerning FMSPs, as these substitutes are produced in compliance with this regulation (Table 2). To assess the adequacy of the protein content of the infant PS, protein equivalents to energy ratios (P.Eq./E) were calculated by dividing the total protein equivalents provided in the nutritional composition per 100 kcal by 100 kcal of product (Table 3). Table 3 also shows the Protein to Energy (P/E) ratios reported in the Commission Delegated Regulation (EU) 2016/127. P.Eq. in PKU protein substitutes cannot be compared to protein content regulated in the Commission Delegated Regulation (EU) 2016/127 also considering that 1 g of amino acids corresponds to 1.2 g of total proteins.

## 3. Results

The results obtained from the comparison of the included seven infant PS for PKU are reported in Table 1. Table 2 reports the food components contained in 100 kcal of infant protein substitute compared to Commission Delegated Regulation (EU) 2016/127 of 25 September 2015 apart from vitamins and minerals that have been compared to Commission Delegated Regulation (EU) 2016/128 of 25 September 2015. In comparing infant protein substitutes for PKU, it is possible to observe that energy content varies from 62 kcal to 69 kcal/100 mL, meeting the target range of 60–70 kcal/100 mL reported by the Commission Delegated Regulation (EU) 2016/127. The average energy density of human milk is 65 kcal/100 mL. The content of protein equivalents (P.Eq.) appears to be heterogeneous, but all the products present a complete amino acid profile. Although Table 3 shows the Protein to Energy (P/E) ratios of infant formula manufactured from cows’ milk or goats’ milk proteins, from soya protein isolates, alone or in a mixture with cows’ milk or goats’ milk proteins, and from protein hydrolysates, P.Eq./E in PKU protein substitutes cannot be compared to protein content regulated in the Commission Delegated Regulation (EU) 2016/128, also because of the equivalence between 1 g of amino acids corresponding to 1.2 g of total proteins. In the Regulation, the L-carnitine content is reported to be at least equal to 1.2 mg/100 kcal for infant formula manufactured from cows’ milk or goats’ milk proteins, from soya protein isolates, alone or in a mixture with cows’ milk or goats’ milk proteins and from protein hydrolysates as well. In our PKU protein substitutes, L-carnitine is always present at a minimum content of 1.49 mg/100 kcal. Considering carbohydrates, they demonstrate an almost similar content among the protein substitutes, all meeting the target range of 9–14 g/100 kcal imposed by the Commission Delegated Regulation (EU) 2016/127. As reported in Table 1, Infant protein substitute 5 has the maximum content of soluble carbohydrates. Only three substitutes (Infant protein substitute 1, Infant protein substitute 3 and Infant protein substitute 6) contain a small amount of fibre. Four formulas contain lactose, but Infant protein substitutes 3 and 6 do not meet the minimum requirement of 4.5 g/100 kcal reported in the Commission Delegated Regulation (EU) 2016/127 (apart from those formulas bearing the statement ‘lactose free’). The statement ‘lactose free’ can be used for infant formula and follow-on formula when the lactose content is not greater than 10 mg/100 kcal. Fructo-oligosaccharides (FOS) and galacto-oligosaccharides (GOS) are found only in two products (Infant protein substitute 1 and Infant protein substitute 6) and, according to the Commission Delegated Regulation (EU) 2016/127, when added, their content shall not exceed 0.8 g/100 mL and the required combination of 90% oligogalactosyl-lactose and 10% high-molecular-weight oligofructosyl-saccharose is respected. Infant protein substitute 3 contains 2’-Fucosyllactose and Lacto-N-tetraose, two of the most abundant human milk oligosaccharides (HMOs) with prebiotic functions. No other product contains compounds with prebiotic activity. The results regarding the content of total fat results tend to be fairly variable among the seven substitutes. Only three companies sent a detailed content of the fatty acids contained in the protein substitutes (Infant protein substitute 1, Infant protein substitute 5 and Infant protein substitute 6). DHA is contained in all the seven Phe-free substitutes and varies from 0.01 g/100 mL (11 mg/100 mL) to 0.02 g/100 mL (20 mg/100 mL) and from 0.02 g/100 kcal to 0.03 g/100 kcal, and to a minimum of 17 mg and a maximum of 29 mg/100 kcal, not respecting the values of 20 mg/100 kcal (minimum) and 50 mg/100 kcal (maximum). Among the seven PKU protein substitutes, only two contain EPA but at substantially negligible levels. Arachidonic acid (ARA) is not mandatory, as stated in Commission Delegated Regulation (EU) 2016/127, but all the products contain it in equal or greater amounts than DHA. Phospholipids in infant formula should not be greater than 2 g/L but, in these protein substitutes for PKU, the value is not reported. Taking into consideration the micronutrients and comparing them with the specific requirements reported in the Commission Delegated Regulation (EU) 2016/128, iron content in the seven products varies satisfying the requirements of this Regulation. Calcium content also varies within the reference values between 50–250 mg/100 kcal. Zinc and selenium are other important micronutrients contained within all the seven products; only Selenium in Infant protein substitute 3 is slightly below the minimum requirement. Looking at vitamin D, its content varies among the products and Infant protein substitutes 1, 3 and 5 do not comply with the required range of 2–3 µg/100 kcal reported in the Regulation. Concerning vitamin B12, all the protein substitutes contain it complying with the Commission Delegated Regulation (EU) 2016/128. Choline is present in all the seven PKU infant protein substitutes, meeting the minimum amount required as reported in the Commission Delegated Regulation (EU) 2016/128. Inositol is present in five out of seven substitutes, while Infant protein substitute 3 contains Myo-inositol. As for nucleotides, the Regulations state that they can be added to formulas, but their total concentration shall not exceed 5 mg/100 kcal. Among the evaluated protein substitutes, Infant protein substitute 3 declares the amount of nucleotides, while Infant protein substitute 5 reports permitted nucleotides among the ingredients, but their amount is not specified.

## 4. Discussion

This is the first review about Phe-free PS available in Italy for infants with PKU, subsequently allowing for detailed assessment of the nutritional composition of these products, which are always used alongside breastfeeding or infant formulas. Among the infant protein substitutes evaluated, the energy values meet the required levels reported in the Commission Delegated Regulation (EU) 2016/127. It should be noted that in PKU, adequate energy intakes are fundamental to prevent catabolism, negative nitrogen balance and elevated Phe levels in blood. Studies have highlighted that formula-fed infants grow at a faster rate over the first year of life compared to breast-fed infants [16], probably because of the higher energy and protein content of formulas compared to breast milk [17]. This rapid growth rate during the first months of life has been associated with a greater chance of developing non-communicable diseases (NCDS) in adulthood [18]. Breast-fed infants usually have a lower protein intake compared to formula-fed infants, leading to important considerations regarding the glucose–insulin axis and prevalence of excess weight in infancy and childhood [19,20]. High-protein intake during infancy may increase weight gain and obesity risk in later years and observational studies have found an increased risk for the development of NCDs, because of the elevated levels of insulin and of insulin-like growth factor-1 (IGF-1) [21,22]. In particular, a protein intake level of 4 g/kg/day is presumably matched with the maximum IGF-1 secretion [23]. Significant attention must be paid to this macronutrient, also considering the emerging hypothesis of NCDs increasing incidence due to the characteristics of PKU long-life diet therapy [24,25]. Considering amino acid needs, an intake of 30–40 kcal/g is usually recommended to guarantee amino acid utilisation [26], corresponding approximately to a P/E ratio of 2.5 g of protein/100 kcal, which is the same maximum protein value reported in the Commission Delegated Regulation (EU) 2016/127 for infant formula manufactured from cows’ milk or goats’ milk proteins. In our study, it was not possible to compare the P.Eq. to proteins in formulas for healthy infants. However, P.Eq. to energy ratios of Phe-free PS were calculated (Table 3), because of their demonstrated role in determining long-term health outcomes also in PKU, considering the restriction of high biological value proteins [27]. In our review, protein intake due to additional breastfeeding or standard infant formula intake must be considered. In fact, the analysed products express the nutritional values of protein substitutes, but are not the only food source for PKU infants, who also consume breast milk or standard infant formulas. In addition, some products have a P.Eq. content exceeding 2.5 g/100 kcal, which is higher than the maximum value of total proteins from infant formula manufactured from cows’ milk or goats’ milk proteins. However, a recent study on PKU showed that a total protein to energy ratio of 3.0–4.5 g total protein/100 kcal/day is safe and can support optimal growth and body composition [27]. Future research should evaluate the adequate P.Eq. to energy ratio in infants with PKU and a legislative reference for the P.Eq. of PS for infants with PKU is required, together with the identification of an optimal P.Eq/E ratio of these products, considering the specific needs of PKUs.

The total fat content complies with the Commission Delegated Regulation (EU) 2016/127. Human milk has an average total fat content of 3.7–9.1 g/100 kcal (around 50% of total energy). Among the most studied functional nutritional components in breast milk there are PUFAs, especially for brain tissue composition and neurodevelopment. ALA is essential in human nutrition as a precursor for n-3 LC-PUFAs: in fact, EPA, docosapentaenoic acid (DPA) and, at a lower amount DHA, are synthesised from ALA. DHA supplementation in infant formula is essential for its potential effect on neurodevelopment [28]. The importance of supplementation in DHA and LC-PUFAs in patients with PKU has been described in various works, which have highlighted the positive influence of these molecules on the neurological development in the first year of life and on maturation of the visual system [12,29]. The Commission Delegated Regulation (EU) 2016/127 states that EPA content, when added, shall not exceed that of DHA, but in our review only two products contain a small amount of EPA. It should also be observed that blood DHA and ARA levels are reduced in PKU patients [30]: low dietary DHA intake could lead to adverse consequences for the central nervous system, contributing to neurodevelopment abnormalities, learning difficulties and behavioural impairment. The use of Phe-free protein substitutes supplemented with adequate LC-PUFAs minimizes the risk of a sub-optimal DHA status and improper neurodevelopment. ARA, although not mandated by the Commission Delegated Regulation (EU) 2016/127, is present in all the seven protein substitutes, highlighting the importance of this acid in infant formulas, along with DHA, as evidenced by recent international evidence [31]. Considering the important role of LC-PUFA in infancy, a recent expert’s opinion [32] recommended the addition of ARA in similar or higher concentrations than those of DHA to infant formulas, or at least similar to the amount of 0.3% of total fatty acids present in human milk, but also possibly reaching 0.64% of total fatty acids owing to its importance for development. This recommendation is particularly important in homozygote infants for haplotype A of human fatty acid desaturase gene (FADS), more widespread among the Latin American population, while in Europe, Asia, and Africa the haplotype D is more prevalent. Indeed, the haplotype A is correlated with variability in LC-PUFAs levels and associated with a lower formation of DHA and, especially, ARA in infancy [32]. Indeed, ARA levels are controlled by FADS1 (delta 5-desaturation) FADS2 (delta 6-desaturation) gene cluster and a variation of approx. 28% for ARA levels can be explained by the single-nucleotide polymorphism (SNP) of the 5-locus and 11-locus haplotypes, possibly increasing the risk for complex diseases (i.e., immunological and neurological diseases) [33]. In our analysis, Infant protein substitute 4 shows higher amounts of DHA than of ARA, while Infant protein substitutes 5 and 6 have the same content of ARA and DHA. For the other four products, the ARA content is higher than the DHA content to varying degrees: from 40% more (Infant protein substitute 3), to 50% more (Infant protein substitutes 1 and 2) to 100% more (Infant protein substitute 7). To recommend the same or a higher ARA concentration than DHA could be likely valid for PKU infants, considering the key role of LC-PUFAs in their neurodevelopment. In fact, while healthy children’s intake of DHA and ARA is assured by the consumption of protein-rich foods after weaning, the intake of DHA and ARA in PKU is guaranteed only from the supplementation of their protein substitutes. Although the supplementation of DHA in PKU is imperative, for ARA, typically low in strict PKU diet, there are no specific recommendations yet.

Regarding carbohydrates, all products analysed meet the required 9–14 g/100 kcal range. Lactose content could be improved in most products, considering that human milk has the highest lactose content (8.2–10.4 g/100 kcal, corresponding to 33–42% of total energy) [34]. Non-digestible carbohydrates in human milk, and in particular oligosaccharides, are one of the three main components of human milk, along with lactose and fats. Several beneficial effects of these molecules are described in the literature: they can reduce the growth and adhesion of various pathogens, prevent pathogen growth and adhesion, reduce inflammatory responses, improve the mucosal barrier function, and may also be involved in infant cognitive development. Moreover, they increase faecal water content and consequently improve gastrointestinal transit and comfort [35]. The Scientific Opinion on the essential composition of infant formulas of the European Food Safety Authority (EFSA) [36] declares the impossibility of adding to them a mixture of oligosaccharides that resemble those in breast milk, because of their variability and complexity. Among the seven PKU protein substitutes, only two of them contain GOS and FOS, both respecting the GOS/FOS 9:1 ratio. According to the EFSA Panel, they are not mandatory components because current evidence is insufficient to declare beneficial effects on infant health acquired from GOS and FOS supplementation. The Commission Delegated Regulation (EU) 2016/127 does not provides any indication of fibre content in formulas for healthy infants. The first study [37] that investigated the addition of prebiotic oligosaccharides (scGOS/lcFOS [9:1 ratio]) to PKU infant formula showed no alterations in Phe control; however, more research is needed to evaluate health benefits. Moreover, the beneficial effect of scGOS/lcFOS [9:1 ratio] on gastrointestinal symptoms, such as constipation, are well known. In addition, it is reported that a different PKU microbiota composition [38] could be potentially linked with an increased risk of developing non-communicable diseases (NCDs), since through the gut–liver axis a pro-inflammatory microbiota profile could lead to a pro-inflammatory status [39]. In our analysis, FOS and GOS are found only in two products and the required ratio of 9:1 for GOS/FOS is respected. Furthermore, only one product contains two of the most important HMOs, 2’-Fucosyllactose and Lacto-N-tetraose, complex glycans with pre-biotic and immunomodulatory functions affecting gut microbiota health from the neonatal period [40]. Breastmilk contains an average of 5–15 g of HMOs per litre, and approximately 200 different structures of HMOs have been identified [41]. These components are well known for their beneficial influence on the immune system as well as on the composition of gut microbiota [42]. No other biotics are added to the analysed PS for PKU infants. Given the known status of gut dysbiosis in PKU following Phe-restricted diet [43], the early modulation of the gut microbiota, also achievable through the addition of biotics (e.g., HMOs, GOS/FOS respecting 9:1 ratio) to infant protein substitutes, could be considered to have beneficial effects on dysbiosis and microbiota–gut–liver–brain axis, although results from clinical intervention studies are needed. A systematic review of the committee on Nutrition of the European Society for Paediatric Gastroenterology, Hepatology, and Nutrition (ESPGHAN) on supplementation of infant formula with probiotics and/or prebiotics highlighted the absence of safety concerns in relation to growth and development, but more experimental research is needed [44].

L-Carnitine is an indispensable nutrient for infants because of their insufficient ability to synthesize it [45]. It acts as a transporter of long-chain fatty acids into the mitochondria where they are oxidised for energy. The minimum content of L-Carnitine (1.2 mg/100 kcal) is mandatory for infant formulas based on cow’s or goat’s milk proteins, on soy proteins and for protein hydrolysates, irrespective of the protein source used [36]. Despite this, all PS for PKU contain this nutrient meeting the minimum requirement and underlining the attention paid to this functional nutrient. Taurine, a non-protein amino acid product formed from the metabolism of methionine and cysteine, is considered essential for normal perinatal development; indeed, infants cannot synthesize it from methionine and cysteine. Taurine is the most prevalent amino acid in human milk. According to observational studies, both preterm and full-term infants with relative taurine deficiency during the neonatal period experience adverse long-term neurodevelopmental outcomes [46]. For this reason, the Codex Alimentarius and EFSA suggest adding taurine to all infant formulas. Although it is not mandatory to add it, five out of seven protein substitutes evaluated contain it. Nucleotides amount is reported only in one of the Infant protein substitutes evaluated, despite representing ∼2% to 5% of the non-protein nitrogen fraction of breast milk as free nucleosides, free nucleotides, RiboNucleic Acid (RNA) and DeoxyriboNucleic Acid (DNA) [47]. Moreover, breastmilk is also a rich source of microRNAs, promoting epigenetic impacts [48], as well as immunoglobulins, cytokines, chemokines, and growth factors [49]; the EFSA Panel decided not to indicate the addition of nucleotides in infant formulas considering the lack of evidence.

### Clinical Considerations for Phe-Free Protein Substitutes for Infants with PKU

All infant formulas must be safe and suitable to satisfy the nutritional requirements and promote appropriate growth and development. In infants with PKU, even if human milk or standard infant formulas can be used alongside infant Phe-free protein substitutes, to date, there are no specific indications about the proportions of the two modalities or the effects on disease control and infant growth [50]. For this reason, infant PS for PKU available on the Italian market should be the best possible alternative to exclusive maternal breastfeeding, providing the nutrient profile as well as the structural and functional effects are as similar as possible to those observed in exclusively breastfeeding or standard infant formulas. The composition of these products is similar to that of internationally available Phe-free PS for PKU, in some cases commercialised under different names. The largest survey conducted on infant feeding practices in PKU throughout Europe [51] showed that over 60% of centres started the dietary treatment at 10 days of age, meeting the European PKU guidelines [3], and highlighting difficulties in breastfeeding maintenance. Among breast-fed infants, the most popular method was to provide a pre-measured dose of Phe-free formula before breastfeeding. Among infants who received formulas, the most frequent practice was to mix standard infant formula together with Phe-free protein substitute. In infants with PKU, Phe tolerance can considerably vary among patients. It has been reported that infants with PKU have a mean intake of Phe between 362 and 464 mL/day of breast milk, providing approximately 3–4 g of intact protein, which is substantially lower than infants on a normal diet [52]. Infants with moderate or severe PKU consume up to 75% of their protein requirements (excluding Phe) from infant protein substitute due to low Phe tolerance, together with an equivalent percentage of their requirements of vitamins and minerals [37].

## 5. Conclusions

The analysis conducted in this study revealed heterogeneity among Phe-free PS suitable for infants with PKU regarding nutritional and functional components. This review did not find any significant shortcomings in these PS, which are safe; nevertheless, their composition might be improved ensuring nutritional quality with a specific regulation, especially concerning protein content. Considering the importance of nutrition in the first 1000 days of life, it is essential to provide PKU newborns with not only the same high-quality amount of macronutrients, micronutrients and functional components of breast milk or formula intended for healthy infants, but a specific regulation that considers the characteristics of PKU infants, who have different nutritional needs compared to those of healthy infants. For this reason, it is necessary to carefully consider the nutritional composition of protein substitutes for infants with PKU not only to ensure the metabolic control of this condition, but to promote proper growth, cognitive neurodevelopment, and immune system health. In addition, carefully addressing requirements regarding the nutritional components of protein substitutes fed to PKU infants could minimize the risk of NCDs onset in adulthood and help to obtain more evidence on the composition of the gut microbiota. Future clinical studies will provide more information on the specific nutrients needed by infants with PKU.

## Figures and Tables

**Table 1:** Food components contained in 100 mL of infant protein substitute. If nutritional data were not present on the nutritional label, the term “Not Declared” (ND) was used.

Content per 100 mL	Unit	Infant Protein Substitute 1	Infant Protein Substitute 2 &	Infant Protein Substitute 3	Infant Protein Substitute 4	Infant Protein Substitute 5	Infant Protein Substitute 6	Infant Protein Substitute 7
Energy	Kj	261	287	282	259	280	293	287
Energy	Kcal	62	68	67	62	67	70	68
Total fats	g	2.99	3.2	3	3.4	3.7	3.45	3.53
Saturated fatty acids	g	0.94	1.4	1.5	1.39	1.76	1.16	0.92
Monounsaturated fatty acids	g	1.52	ND	1.3	1.26	1.24	1.7	1.76
Polyunsaturated fatty acids	g	0.52	ND	0.6	0.73	0.67	0.6	0.63
Caproic acid	g	0	ND	ND	0	0	0	ND
Caprylic acid	g	0.06	ND	ND	ND	0.05	0.06	ND
Capric acid	g	0.05	ND	ND	ND	0.04	0.05	ND
Lauric acid	g	0.41	ND	ND	ND	0.29	0.37	ND
Myristic acid	g	0.15	ND	ND	ND	0.14	0.16	ND
Palmitic acid	g	0.18	ND	ND	ND	0.89	0.24	ND
Palmitoleic acid	g	ND	ND	ND	ND	0.01	0	ND
Stearic acid	g	0.08	ND	ND	ND	0.2	0.16	ND
Oleic acid	g	1.51	ND	ND	ND	1.15	1.61	ND
Linoleic acid	g	0.42	0.4	0.5	0.63	0.56	0.48	0.54
Alpha-linolenic acid	g	0.06	0.04	0.05	0.06	0.05	0.05	0.05
Docosahexaenoic acid (DHA)	g (mg)	0.01 (12)	0.02 (20)	0.02 (16)	0.01 (13)	0.01 (11)	0.02 (18)	0.01 (14)
Arachidonic acid	g (mg)	0.02 (18)	0.03 (30)	0.02 (22)	0.01 (11)	0.01 (11)	0.02 (18)	0.03 (28)
Eicosapentaenoic acid	g (mg)	ND	ND	ND	ND	0 (2,46)	0 (0,05)	ND
Cholesterol	g	ND	ND	ND	ND	0	ND	ND
Carbohydrates	g	6.76	7.9	8	6.43	7.25	7.52	7.19
Soluble carbohydrates	g	0.6	4.2	3.5	4.41	5.36	1.11	0.71
Starch	g	ND	ND	1	ND	ND	ND	ND
Polyols	g	ND	ND	ND	ND	ND	ND	ND
Fructose	g	ND	ND	ND	ND	0	ND	ND
Maltodextrins	g	6.16	ND	3.5	2.02	ND	ND	ND
Lactose	g	ND	ND	3	4.28	5.4	0.26	ND
D-mannose	g	ND	ND	ND	ND	ND	ND	ND
Dietary fibre	g	0.64	0	0.14	ND	0	0.56	ND
2’-Fucosyllactose	g			0.1				
Lacto-N-tetraose	g			0.05				
GOS	g	0.57	ND	ND	ND	ND	0.48	ND
FOS	g	0.07	ND	ND	ND	ND	0.08	ND
Protein equivalents	g	1.76	2	1.6	1.51	1.25	1.97	2.02
L-Alanine	g	0.08	0.12	0.07	0.1	0.07	0.09	0.08
L-Arginine	g	0.13	0.15	0.07	0.08	0.06	0.16	0.14
L-Aspartic acid	g	0.20	0.24	0.15	0.14	0.13	0.15	0.22
L-Cystine	g	0.01	0.06	0.04	0.04	0.04	0.06	0.05
L-Glutamine	g	0.16	ND	ND	0.04	ND	0.21	0.16
Glycine	g	0.2	0.24	0.06	0.1	0.04	0.14	0.21
L-Histidine	g	0.08	0.09	0.06	0.04	0.04	0.09	0.08
L-Isoleucine	g	0.12	0.16	0.11	0.09	0.1	0.14	0.15
L-Leucine	g	0.22	0.25	0.18	0.15	0.16	0.24	0.23
L-Lysine	g	0.16	0.17	0.13	0.13	0.11	0.17	0.15
L-Methionine	g	0.04	0.04	0.03	0.03	0.04	0.04	0.04
L-Phenylalanine	g	0	0	0	<0.01	ND	ND	ND
L-Proline	g	0.14	0.17	0.15	0.17	0.15	0.17	0.15
L-Serine	g	0.09	0.11	0.1	0.12	0.08	0.11	0.1
Taurine	mg	5.20	4.27	5	ND	5.65	3.45	ND
L-Threonine	g	0.12	0.16	0.08	0.12	0.08	0.12	0.15
L-Tryptophan	g	0.04	0.05	0.04	0.03	0.03	0.05	0.05
L-Tyrosine	g	0.2	0.24	0.17	0.18	0.12	0.22	0.22
L-Valine	g	0.14	0.18	0.11	0.07	0.1	0.16	0.17
L-Carnitine	mg	1.76	2.1	1.5	0.92	1.32	1.52	2.12
L-Glutamic acid	g	0	ND	0.25	0.17	0.25	ND	ND
Nucleotides	mg			3				
Vitamin A	µg RE	53.3	75	63	71.95	60.46	61.2	64.86
Vitamin D3	µg	1.16	1.7	1.3	1.26	1.03	1.68	1.62
Vitamin E	mg α-TE	1.16	0.7	0.4	1.12	0.78	1.73	0.99
Vitamin C	mg	6.97	15	8	11.47	6.86	7.34	8.6
Vitamin K1	µg	4.64	6	0.9	2.9	2.73	5.6	5.64
Vitamin K2 (MK-7)	µg	0	ND	ND	ND	ND	ND	ND
Vitamin B1, Thiamin	mg	0.07	0.05	0.04	0.06	0.04	0.08	0.06
Vitamin B2, Riboflavin	mg	0.09	0.1	0.05	0.11	0.05	0.08	0.07
Vitamin B3, Niacin	mg	0.7	0.9	0.5	0.59	1.03	0.35	0.47
Niacin NE		ND	ND	ND	ND	ND	ND	ND
Vitamin B6	mg	0.07	0.04	0.03	0.07	0.03	0.08	0.06
Total folate	µg	6.99	8	9	10.84	7.77	8.25	10.86
Vitamin B12	µg	0.17	0.23	0.13	0.14	0.12	0.18	0.17
Biotin	µg	5.81	1.7	0.7	1.39	1.21	2.73	2.68
Pantothenic acid	mg	0.7	0.4	0.3	0.5	0.3	0.42	0.35
Choline	mg	25.09	24	17	15.75	16.9	21.9	21.43
Calcium	mg	58.11	60	34	61.36	50.16	61.5	56.4
Chromium	µg	1.69	2	2.2	1.23	2.07	2.1	2.04
Copper	mg	0.05	0.6	0.08	0.04	0.04	0.06	0.06
Iodine	µg	11.05	14	10	11.97	7.08	14.69	14.95
Iron	mg	0.7	0.8	0.8	0.65	0.78	1.19	0.82
Magnesium	mg	5.72	6	7	9.2	5.02	8.73	6.35
Manganese	mg	0.03	0.045	0.06	0.03	0.05	0	0.04
Molybdenum	µg	1.17	5	2	1.51	2.63	1.82	2.26
Phosphorus	mg	37.7	45	28	37.17	30.36	45	42.3
Selenium	µg	2.86	3	2	3.15	2.09	2.66	2.96
Zinc	mg	0.59	0.9	0.34	0.61	0.47	0.84	0.49
Potassium	mg	58.11	75	63	55.31	65.47	75.75	71.21
Sodium	mg	26.13	24	21	22.81	18.35	28.65	26.79
Chloride	mg	40.82	55	46	48.01	36.96	53.25	51.47
Inositol	mg	4.64	ND	ND	12.6	11.62	14.69	8.46
Myo-inositol	mg	ND	ND	7	ND	ND	ND	ND
Lutein	mg	0	ND	ND	ND	ND	ND	ND
Fluoride	mg	0	ND	0.04	ND	0.01	ND	ND
Salt	g	0.07	0.06	0.006	0.06	0.05	0.07	0.07

**Explanation of infant protein substitutes:** Infant protein substitute 1: DMF Antifen Start; Infant protein substitute 2: Medifood Afenil 1; Infant protein substitute 3: MamoXi XPhe Infant MixLCP; Infant protein substitute 4: Mevalia PKU Balance Complete 0–1; Infant protein substitute 5: Nutricia Milupa PKU 1-Mix; Infant protein substitute 6: Nutricia PKU Anamix Infant; Infant protein substitute 7: Vitaflo PKU Start. &: Ready-to-use Phe-free drink.

**Table 2:** Food components contained in 100 kcal of infant protein substitute compared to Commission Delegated Regulation (EU) 2016/127 of 25 September 2015 and to Commission Delegated Regulation (EU) 2016/128 of 25 September 2015 for vitamins and minerals. If nutritional data were not present on the nutritional label, the term “Not Declared” (ND) was used. A “!” is used when the value does not comply with the Regulation.

**Content per 100 mL**	**Unit**	**Infant Protein Substitute 1**	**Infant Protein Substitute 2** **&**	**Infant Protein Substitute 3**	**Infant Protein Substitute 4**	**Infant Protein Substitute 5**	**Infant Protein Substitute 6**	**Infant Protein Substitute 7**	**MIN** **EU 2016/127**	**MAX** **EU 2016/127**
		From birth	From birth	From birth	From birth	From birth	From birth	From birth		
Energy	Kj	261	287	282	259	280	293	287	250	293
Energy	Kcal	62	68	67	62	67	70	69	60	70
**Content per 100 kcal**	**Unit**	**Infant protein substitute 1**	**Infant protein substitute 2** **&**	**Infant protein substitute 3**	**Infant protein substitute 4**	**Infant protein substitute 5**	**Infant protein substitute 6**	**Infant protein substitute 7**	**MIN** **EU 2016/127**	**MAX** **EU 2016/127**
Total fats	g	4.79	4.71	4.48	5.49	5.52	4.94	5.14	4.4	6.0
Saturated fatty acids	g	1.50	2.06	2.24	2.24	2.62	1.65	1.34		
Monounsaturated fatty acids	g	2.44	ND	1.94	2.03	1.85	2.42	2.57		
Polyunsaturated fatty acids	g	0.83	ND	0.90	1.18	1.01	0.86	0.93		
Caproic acid	g	0.01	ND	ND	0.00	0	0.01	ND		
Caprylic acid	g	0.09	ND	ND	ND	0.07	0.09	ND		
Capric acid	g	0.07	ND	ND	ND	0.06	0.07	ND		
Lauric acid	g	0.66	ND	ND	ND	0.44	0.53	ND		
Myristic acid	g	0.24	ND	ND	ND	0.21	0.23	ND		
Palmitic acid	g	0.29	ND	ND	ND	1.33	0.34	ND		
Palmitoleic acid	g	ND	ND	ND	ND	0.01	0	ND		
Stearic acid	g	0.13	ND	ND	ND	0.29	0.23	ND		
Oleic acid	g	2.42	ND	ND	ND	1.72	2.3	ND		
Linoleic acid	g	0.67	0.59	0.75	1.02	0.83	0.69	0.78	0.5	1.2
Alpha-linolenic acid	g	0.1	0.06	0.07	0.1	0.07	0.07	0.07	0.05	0.1
Docosahexaenoic acid (DHA)	g (mg)	0.02 (19)	0.03 (29)	0.02 (22)	0.02 (21)	0.02 (17)	0.03 (26)	0.02 (20)	0.02 (20)	0.05 (50)
Arachidonic acid	g (mg)	0.03 (29)	0.04 (40)	0.03 (33)	0.02 (18)	0.02 (17)	0.03 (26)	0.04 (41)		
Eicosapentaenoic acid	g (mg)	ND	ND	ND	ND	0 (0.48)	0 (0.01)	ND		
Cholesterol	g	ND	ND	ND	ND	0.01	ND	ND		
**Content per 100 kcal**	**Unit**	**Infant protein substitute 1**	**Infant protein substitute 2** **&**	**Infant protein substitute 3**	**Infant protein substitute 4**	**Infant protein substitute 5**	**Infant protein substitute 6**	**Infant protein substitute 7**	**MIN** **EU 2016/127**	**MAX** **EU 2016/127**
Carbohydrates	g	10.83	11.62	11.94	10.37	10.83	10.75	10.49	9	14
Soluble carbohydrates	g	0.96	6.18	5.22	7.11	8.01	1.59	1.03		
Starch	g	ND	ND	1.49	ND	0	0	0	/	2
Polyols	g	ND	ND	ND	ND	ND	ND	ND		
Fructose	g	ND	ND	ND	ND	0	/	ND		
Maltodextrins	g	9.88	ND	5.22	3.25	ND	ND	ND		
Lactose	g	ND	ND	4.48	6.91	8.07	0.36	ND	4.5	/
D-mannose	g	ND	ND	ND	ND	ND	ND	ND		
Dietary fibre	g	1.02	0	0.21	ND	0	0.79	ND		
2’-Fucosyllactose	g			0.15						
Lacto-N-tetraose	g			0.07						
GOS	g	0.92	ND	ND	ND	ND	0.69	ND		^
FOS	g	0.1	ND	ND	ND	ND	0.11	ND		^
**Content per 100 mL**	**Unit**	**Infant protein substitute 1**	**Infant protein substitute 2** **&**	**Infant protein substitute 3**	**Infant protein substitute 4**	**Infant protein substitute 5**	**Infant protein substitute 6**	**Infant protein substitute 7**		
GOS	g	0.57	ND	ND	ND	ND	0.48	ND		^
FOS	g	0.07	ND	ND	ND	ND	0.08	ND		^
**Content per 100 kcal**	**Unit**	**Infant protein substitute 1**	**Infant protein substitute 2** **&**	**Infant protein substitute 3**	**Infant protein substitute 4**	**Infant protein substitute 5**	**Infant protein substitute 6**	**Infant protein substitute 7**	**MIN**	**MAX**
Protein equivalents	g	2.81	2.94	2.39	2.44	1.87	2.81	2.94		
**Content per 100 kcal**	**Unit**	**Infant protein substitute 1**	**Infant protein substitute 2** **&**	**Infant protein substitute 3**	**Infant protein substitute 4**	**Infant protein substitute 5**	**Infant protein substitute 6**	**Infant protein substitute 7**	**MIN** **EU 2016/127**	**MAX** **EU 2016/127**
L-Alanine	g	0.13	0.18	0.10	0.16	0.10	0.13	0.12		
L-Arginine	g	0.21	0.22	0.10	0.13	0.09	0.23	0.20		
L-Aspartic acid	g	0.31	0.35	0.22	0.23	0.20	0.22	0.31		
L-Cystine	g	0.02	0.09	0.06	0.06	0.06	0.09	0.08		
L-Glutamine	g	0.25	ND	ND	0.06	ND	0.30	0.23		
Glycine	g	0.31	0.35	0.09	0.16	0.06	0.20	0.31		
L-Histidine	g	0.13	0.13	0.09	0.06	0.06	0.13	0.12		
L-Isoleucine	g	0.19	0.24	0.16	0.14	0.14	0.20	0.21		
L-Leucine	g	0.35	0.37	0.27	0.24	0.24	0.35	0.34		
L-Lysine	g	0.25	0.25	0.19	0.21	0.16	0.24	0.22		
L-Methionine	g	0.06	0.06	0.04	0.05	0.06	0.06	0.06		
L-Phenylalanine	g	0	0	0	<0.01	ND	ND	ND		
L-Proline	g	0.23	0.25	0.22	0.28	0.22	0.25	0.22		
L-Serine	g	0.15	0.16	0.15	0.20	0.12	0.15	0.14		
Taurine	mg	8.33	6.28	7.46	ND	8.44	4.94	ND		12
L-Threonine	g	0.19	0.24	0.12	0.19	0.11	0.17	0.22		
L-Tryptophan	g	0.06	0.07	0.06	0.05	0.05	0.07	0.07		
L-Tyrosine	g	0.31	0.35	0.25	0.29	0.18	0.31	0.32		
L-Valine	g	0.23	0.26	0.16	0.12	0.15	0.22	0.25		
L-Carnitine	mg	2.81	3.09	2.24	1.49	1.97	2.17	3.09	1.2	
L-Glutamic acid	g	0	ND	0.37	0.27	0.37	ND	ND		
Nucleotides	mg			4.48						5
**Content per 100 kcal**	**Unit**	**Infant protein substitute 1**	**Infant protein substitute 2** **&**	**Infant protein substitute 3**	**Infant protein substitute 4**	**Infant protein substitute 5**	**Infant protein substitute 6**	**Infant protein substitute 7**	**MIN** **EU 2016/128**	**MAX** **EU 2016/128**
Vitamin A	µg RE	85.42	110.29	94.03	116.06	90.34	87.55	94.65	70	180
Vitamin D3	µg	1.85 !	2.5	1.94!	2.03	1.55!	2.4	2.37	2	3
Vitamin E	mg α-TE	1.85	1.03	0.60	1.81	1.16	2.47	1.44	0.6	5
Vitamin C	mg	11.17	22.06	11.94	18.50	10.26	10.49	12.55	4	30
Vitamin K1	µg	7.44	8.82	1.34	4.67	4.08	8.00	8.23	1	25
Vitamin K2 (MK-7)	µg	0	ND	ND	ND	ND	ND	ND		
Vitamin B1, Thiamin	mg	0.1	0.07	0.06	0.09	0.07	0.11	0.08	0.04	0.3
Vitamin B2, Riboflavin	mg	0.15	0.15	0.07	0.18	0.08	0.11	0.10	0.06	0.45
Vitamin B3, Niacin	mg	1.13	1.32	0.75	0.95	1.53	0.5	0.68	0.4	3
Niacin NE		ND	ND	ND	ND	ND	ND	ND	0.4	3
Vitamin B6	mg	0.1	0.06	0.04	0.12	0.05	0.11	0.08	0.02	0.3
Total folate $	µg	11.21	11.76	13.43	17.48	11.62	11.8	15.84	9 (DFE = 15)	28.56 (DFE = 47.6)
Vitamin B12	µg	0.27	0.34	0.19	0.23	0.18	0.26	0.25	0.1	0.5
Biotin	µg	9.31	2.5	1.04	2.24	1.80	3.91	3.91	1	20
Pantothenic acid	mg	1.13	0.59	0.45	0.81	0.45	0.6	0.51	0.4	2
Choline	mg	40.21	35.29	25.37	25.41	25.25	31.33	31.28	25	50
Calcium *	mg	93.13	88.24	50.75	98.98	74.95	87.98	82.30	50	250
Chromium	µg	2.71	2.94	3.28	1.98	3.10	3	2.98	/	10
Copper	mg	0.08	0.88	0.12	0.07	0.07	0.09	0.08	0.06	0.12
Iodine	µg	17.71	20.59	14.93!	19.31	10.57!	21.01	21.81	15	35
Iron	mg	1.13	1.18	1.19	1.05	1.17	1.7	1.19	0.3	2.5
Magnesium	mg	9.17	8.82	10.45	14.84	7.5	12.49	9.26	5	15
Manganese	mg	0.04	0.07	0.09	0.05	0.07	0.01	0.05	0.001	0.1
Molybdenum	µg	1.88	7.35	2.99	2.44	3.93	2.60	3.29	/	14
Phosphorus	mg	60.42	66.18	41.79	59.96	45.36	64.38	61.73	25	100
Selenium	µg	4.58	4.41	2.99	5.08	3.12	3.80	4.32	3	8.6
Zinc	mg	0.94	1.32	0.51	0.99	0.71	1.20	0.72	0.5	2.4
Potassium	mg	93.13	110.29	94.03	89.23	97.83	108.37	103.91	80	160
Sodium	mg	41.88	35.29	31.34	36.79	27.42	40.99	39.09	25	60
Chloride	mg	65.42	80.88	68.66	77.44	55.23	76.18	75.1	60	160
Inositol	mg	7.44	ND	ND	20.33	17.36	21.01	12.35	4	40
Myo-inositol	mg	ND	ND	10.45	ND	ND	ND	ND		
Lutein	mg	0	ND	ND	ND	ND	ND	ND		
Fluoride	mg	0	ND	0.07	ND	0.01	ND	ND	/	0.2
Salt	g	0.10	0.09	0.09	0.09	0.07	0.1	0.1		

^ FOS and GOS may be added to infant formula, in which case their content shall not exceed: 0.8 g/100 mL in a combination of 90% oligogalactosyl-lactose and 10% high-molecular-weight oligofructosyl-saccharose (Commission Delegated Regulation (EU) 2016/127 of 25 September 2015). **Explanation of infant protein substitutes:** Infant protein substitute 1: DMF Antifen Start; Infant protein substitute 2: Medifood Afenil 1; Infant protein substitute 3: MamoXi XPhe Infant MixLCP; Infant protein substitute 4: Mevalia PKU Balance Complete 0–1; Infant protein substitute 5: Nutricia Milupa PKU 1-Mix; Infant protein substitute 6: Nutricia PKU Anamix Infant; Infant protein substitute 7: Vitaflo PKU Start. & Ready-to-use Phe-free drink. * The calcium:available phosphorus molar ratio shall not be less than 1 nor greater than 2. $ Dietary folate equivalent: 1 μg DFE = 1 μg food folate = 0.6 μg folic acid from food for special medical purposes.

**Table 3:** Protein equivalents (P.Eq.) to energy ratio for all the infant protein substitutes, compared to Protein to Energy (P/E) ratios reported in the Commission Delegated Regulation (EU) 2016/127 of 25 September 2015.

Protein Equivalents (P.Eq.) to Energy Ratio	Infant Protein Substitute 1	Infant Protein Substitute 2 &	Infant Protein Substitute 3	Infant Protein Substitute 4	Infant Protein Substitute 5	Infant Protein Substitute 6	Infant Protein Substitute 7	MIN P/E EU 2016/127	MAX P/E EU 2016/127
	2.81 g/100 kcal	2.94 g/100 kcal	2.39 g/100 kcal	2.44 g/100 kcal	1.87 g/100 kcal	2.81 g/100 kcal	2.94 g/100 kcal	1.8 g/100 kcal *	2.5 g/100 kcal *
								2.25 g/100 kcal @	2.8 g/100 kcal @
								1.86 g/100 kcal #	2.8 g/100 kcal #

**Explanation of infant protein substitutes**: Infant protein substitute 1: DMF Antifen Start; Infant protein substitute 2: Medifood Afenil 1; Infant protein substitute 3: MamoXi XPhe Infant MixLCP; Infant protein substitute 4: Mevalia PKU Balance Complete 0–1; Infant protein substitute 5: Nutricia Milupa PKU 1-Mix; Infant protein substitute 6: Nutricia PKU Anamix Infant; Infant protein substitute 7: Vitaflo PKU Start. & Ready-to-use Phe-free drink. * Infant formula manufactured from cows’ milk or goats’ milk proteins; @ Infant formula manufactured from soya protein isolates, alone or in a mixture with cows’ milk or goats’ milk proteins; # Infant formula manufactured from protein hydrolysates.

## Data Availability

Not applicable.

## Appendix A. Specific Compositional Nutritional Requirements for Phe-Free Protein Substitutes, Laid Down Commission Delegated Regulation (EU) 2016/127 (Table A1) and by Commission Delegated Regulation (EU) 2016/128 (Table A2)

**Table A1:** Nutritional requirements of infant formula reported in the Commission Delegated Regulation (EU) 2016/127.

Content per 100 mL	Unit	MIN	MAX
Energy	kcal	60	70
L-carnitine	mg	1.2	
Taurine !	mg		12
Choline	mg	25	50
Lipids	g	4.4	6
Linoleic acid	mg	500	1200
Alpha-linolenic acid	mg	50	100
Docosahexaenoic acid (DHA)	mg	20	50
Eicosapentenoic acid (EPA) #	mg		
Phospholipids (g)/L	g		2
Inositol	mg	4	40
Carbohydrates	g	9	14
Lactose £	g	4.5	
Fructo-oligosaccharides (FOS) $	g		
Galacto-oligosaccharides (GOS) $	g		
Nucleotides ^	mg		5

! The amount of taurine that may be added to infant formulas should not exceed 12 mg/100 kcal. # It is recommended that levels of EPA should not exceed those of DHA. £ Except for formulas mainly based on soy proteins and those specifically created without lactose for dedicated uses. $ FOS and GOS may be added but their quantity must be less than 0.8 g/100 mL and must be kept in combination of 90% GOS and 10% FOS. ^ If added, the total concentration of nucleotides shall not exceed 5 mg/100 kcal.

**Table A2:** Values for vitamins and minerals in FSMPs developed to satisfy the nutritional requirements of infants reported in the Commission Delegated Regulation (EU) 2016/128.

Content per 100 kcal	Unit	MIN	MAX
Vitamin A	mg-RE	70	180
Vitamin D	mg	2	3
Vitamin K	mg	1	25
Vitamin C	mg	4	30
Vitamin B1, Thiamin	mg	0.04	0.3
Vitamin B2, Riboflavin	mg	0.06	0.45
Vitamin B6	mg	0.02	0.3
Niacin	mg	0.4	3
Total Folate $	mg	9 (DFE = 15)	28.56 (DFE = 47.6)
Vitamin B12	mg	0.1	0.5
Pantothenic acid	mg	0.4	2
Biotin	mg	1	20
Vitamin E	mg α-TE	0.6	5
Sodium	mg	25	60
Chloride	mg	60	160
Potassium	mg	80	160
Calcium *	mg	50	250
Phosphorus	mg	25	100
Magnesium	mg	5	15
Iron	mg	0.3	2.5
Zinc	mg	0.5	2.4
Copper	mg	0.06	0.12
Iodine	mg	15	35
Selenium	mg	3	8.6
Manganese	mg	0.001	0.1
Chromium	mg		10
Molybdenum	mg		14
Fluoride	mg		0.2

* The calcium:available phosphorus molar ratio shall not be less than 1 nor greater than 2. $ Dietary folate equivalent: 1 μg DFE = 1 μg food folate = 0.6 μg folic acid from FSMPs.
